# Molecular Hydrogen: Is This a Viable New Treatment for Plants in the UK?

**DOI:** 10.3390/plants10112270

**Published:** 2021-10-22

**Authors:** John T. Hancock, Tyler W. LeBaron, Jennifer May, Adam Thomas, Grace Russell

**Affiliations:** 1 Department of Applied Sciences, University of the West of England, Bristol BS16 1QY, UK; Jennifer2.May@uwe.ac.uk (J.M.); Adam7.Thomas@uwe.ac.uk (A.T.); Grace.Russell@uwe.ac.uk (G.R.); 2 Centre of Experimental Medicine, Institute for Heart Research, Slovak Academy of Sciences, Faculty of Natural Sciences of Comenius University, 84104 Bratislava, Slovakia; sci_ty7@yahoo.com; 3 Molecular Hydrogen Institute, Enoch, UT 84721, USA; 4 Department of Kinesiology and Outdoor Recreation, Southern Utah University, Cedar City, UT 84720, USA

**Keywords:** heme, heme oxygenase, hydrogen gas, nitric oxide, postharvest, reactive oxygen species, redox, stress responses

## Abstract

Despite being trialed in other regions of the world, the use of molecular hydrogen (H_2_) for enhanced plant growth and the postharvest storage of crops has yet to be widely accepted in the UK. The evidence that the treatment of plants and plant products with H_2_ alleviates plant stress and slows crop senescence continues to grow. Many of these effects appear to be mediated by the alteration of the antioxidant capacity of plant cells. Some effects seem to involve heme oxygenase, whilst the reduction in the prosthetic group Fe^3+^ is also suggested as a mechanism. Although it is difficult to use as a gaseous treatment in a field setting, the use of hydrogen-rich water (HRW) has the potential to be of significant benefit to agricultural practices. However, the use of H_2_ in agriculture will only be adopted if the benefits outweigh the production and application costs. HRW is safe and relatively easy to use. If H_2_ gas or HRW are utilized in other countries for agricultural purposes, it is tempting to suggest that they could also be widely used in the UK in the future, particularly for postharvest storage, thus reducing food waste.

## 1. Introduction

The use of molecular hydrogen (H_2_) in agriculture is suggested to have a bright future [1,2]. However, its wide use in agricultural practices, including in the UK, has yet to gain traction despite increasing evidence of its benefits (discussed in Section 3 and Section 4). 

The evidence that H_2_ has effects on biological systems is growing. This is exemplified in the biomedical field, where H_2_ research suggests that this diatomic gas may have an important future as a medical therapy [3,4], and there has been a growing body of evidence for its beneficial effects in the biomedical field for some time. H_2_-based treatments are reported to be advantageous for a range of human diseases, including COVID-19 [5,6,7], where the inhalation of H_2_ was the subject of a recent clinical trial [8]. Other conditions for which H_2_ is shown to have positive effects include neurodegenerative diseases [9,10], such as Parkinson’s Disease [11], as well as rheumatoid arthritis [12], type-2 diabetes [13], metabolic syndrome [14], Hepatitis B [15] and cancer [16]. Therefore, there seems to be little doubt that the treatment of animal cells with H_2_, including human cells, has significant effects, hence it is being considered as a future therapy [3,4]. As discussed below, much of the discussion regarding how H_2_ interacts with human and animal cells is translational to plant cells. Therefore, a full understanding of how H_2_ functions will be beneficial to many aspects of biological sciences, including informing how H_2_ could be used in agricultural practices. For the adoption of H_2_-based treatments in agriculture there needs to be a benefit which outweighs the costs. This is not such a relevant issue for treating human conditions but, as H_2_ production for medical use is developed, it may lead to a better cost–benefit ratio, and therefore be viable for the adoption of some plant treatments. Alternatively, if the use of H_2_ in agriculture demonstrates clear benefits, then the cost of using H_2_ for medical applications may also be reduced. Critically, the use of H_2_ in a clinical setting suggests that it is safe for human consumption, which is an important issue if it is to be used to increase the shelf life of food products. 

With such a body of evidence reporting that H_2_ has significant beneficial effects in humans, the positive effects seen in plants are not unexpected. Such effects are mainly seen when plants are exposed to stress, which aligns with the reports in humans, where H_2_ is being trialed for use in degenerative conditions [9,10,11] and for numerous diseases [5,6,7]. In plants, H_2_ has been suggested as a treatment which should be adopted by agricultural practices [17], although this has not yet happened in the UK. For H_2_ to be used widely anywhere in the world, the cost of its application must be less than its benefits. For this to be properly assessed, agricultural trials would need to be carefully monitored. Since such trials have yet to take place, it is understandable that there is no urgency for the adoption of H_2_-based applications in plants on a large scale. Therefore, at present all the data in the literature are from small trials and laboratory-based experiments. Here, we attempt to pool these data together and discuss the pitfalls and potential benefits of H_2_ use on plants.

On a cellular level, plants can be exposed to H_2_ by a variety of means. For example, enzymes such as hydrogenases can generate H_2_ endogenously, as reviewed in [18]. Although not a higher plant, the H_2_ production by the algae *Chlamydomonas reinhardtii* is well characterized [19]. The generation of H_2_ by this alga is so great that it is recommended as a source of H_2_ for use as a biofuel [20], although some manipulation of the intracellular mechanisms is needed for the H_2_ production to be sustained [21]. Such a model of cellular generation of H_2_ is important for understanding the biochemistry of H_2_ in higher plants, but if the use of this algae is ever commercially viable as a fuel it has the potential to be an exogenous source of H_2_, which can be exploited in the future for agricultural purposes. Plants may also be exposed to H_2_ from external sources. This could be from the microflora in the locale of the plant [22] or from certain treatments, which may be useful in agriculture. Here, many of the physiological effects of H_2_ are discussed, along with the possible cellular mechanisms of action. Finally, the application and use of H_2_ for enhanced plant growth and crop production is also discussed.

The activities of plant cells are determined by a suite of control mechanisms, including the arrival of signaling molecules from endogenous and exogenous sources. Among these molecules are several which are gaseous in nature and are termed gasotransmitters. These include ethylene [23], nitric oxide (NO) [24] and hydrogen sulfide (H_2_S) [25,26]; the actions of these compounds are the focus of intense investigation. NO activity has been recognized in plants for more than forty years [27] but, more recently, it was acknowledged that H_2_S [28] and H_2_ are also important compounds [29]. However, it is unlikely that such molecules act alone. There are many interactions between these signaling molecules, partly because of their reactivity similarities. Similar to the reactive oxygen species (ROS), both NO and H_2_S can react with the thiol groups [30,31]. These signaling molecules also seem to modulate the antioxidant capacity of cells [32,33], as well as react with one another. The interactions of ROS, NO and H_2_S have recently been extensively reviewed [26,34,35,36,37]. However, what is pertinent here is that H_2_ is thought to be part of this web of small, often gaseous, but relatively reactive signaling molecules, and therefore modulating its presence and concentration may have profound and positive effects on plants [17]. H_2_ is shown to affect the growth and fitness of plants [38] with such favorable effects mediated through the levels of phytohormones [39] such as gibberellin and auxin [40]. As mentioned above, this has parallels with animal studies; H_2_ was shown to increase the cellular antioxidant capacity in both animals and plants [41,42]. However, in both plants and animals, one of the fundamental conundrums regarding the use of H_2_ in biological systems is understanding its function. As alluded to previously [43] it is hard to envisage how H_2_ would be sensed by a classical receptor protein, as it is so small and neutral. H_2_ is also relatively inert, so it is unlikely to react directly with amino acids, such as cysteine thiol groups, an action common to ROS, NO and H_2_S [44]. Certainly, to date, no specific receptor has been identified, and no thiol modification for H_2_ application has been reported in any cell type studied, either in animals or plants. Therefore, there must be another explanation of how H_2_ interacts with cellular components if its effects are causational.

Early research suggests that H_2_ has a selective, reductive activity against ROS and reactive nitrogen species (RNS), especially against the hydroxyl radical (∙OH) and peroxynitrite (ONOO^−^) [45], but this is disputed when the kinetics of the reactions are considered [46,47]. One study suggested that the Fe^3+^ of prosthetic groups could be the target, but limited examples were used, and thus no evidence for this was presented [46]. In plants there are many heme groups involved in respiration and photosynthesis, as well as in ROS and NO metabolism, all which may be potential H_2_ targets which could account for the effects seen in H_2_ application [46]. The possibilities of such reactions from a redox potential point of view, with no allowance for kinetics, were recently discussed [48]. It may be possible for some heme prosthetic groups to be targets, but the experimental data need to be gathered to support this view. Alternatively, it was suggested that the hydrogen spin states should also be considered [49]. 

There is mounting evidence that the antioxidant capacity of cells is altered by H_2_ administration [41,42], but the direct link back to the molecular targets of H_2_ has yet to be established. There are reports of heme oxygenase-1 (HO-1) mediating the H_2_ effects, which account for some of the responses seen in animals [50,51] and plants [52,53]. In fungi, glutathione peroxidase, a selenium-based enzyme, was shown to meditate hydrogen-rich water (HRW) effects [54], although how H_2_ interacted in this case was not specifically determined. 

Regardless of the limited understanding of how H_2_ functions in cells, there is a growing body of evidence which describes H_2_ as eliciting positive responses in plants; therefore, its use in agriculture should be considered. The application of molecular hydrogen appears to be safe, leaving no toxic by-products, and has beneficial effects for plants and plant materials such as crops. Undoubtedly, the direct molecular interactions will be understood in time, but this should not be the reason to delay its introduction into agricultural practices.

## 2. H_2_ as a Future Plant Treatment

For H_2_ to be useful for any aspects of agricultural practices it must be safe, cost-effective and easy to apply. Indeed, there are other gaseous treatments, including some of the gasotransmitters discussed above. For instance, NO and donor molecules which release NO are suggested to be useful [55], as is H_2_S [56], including the use in postharvest storage [57]. However, both NO and H_2_S are inherently toxic [58,59], whereas there are no reports of H_2_ toxicity to date, although, to confirm this, clinical trials assessing H_2_ toxicity are planned [60]. However, as mentioned, the use of H_2_ in a clinical setting suggests that it is safe, and can be used in food products.

Accordingly, for H_2_ to have an advantage over other gaseous treatments, it needs to have to a more significant effect and be safer and easier to use. The use of H_2_ also needs to cost less than the amount of enhanced profits that it might elicit. It is hard to envisage a widespread usage for H_2_ in field agriculture in its gaseous form, partly as it is highly flammable [61,62], and thus a potential safety hazard. However, in the biomedical field, gaseous application is an option as such substances can be directly breathed in, with hydrogen/air, or hydrogen/oxygen mixtures delivered via a nebulizer, for example [63]. It can also be administered at concentrations below the flammability level (<4.6%) and still exert therapeutic effects. Furthermore, hydrogen gas is lighter than air, as exemplified by its use in aircraft [64], so it is difficult to use as a treatment for ground-level plants. In the biomedical field this can be overcome by inhalation [65], but this is not an option for plants unless H_2_ is used within a closed environment, which would require additional safety protocols. The volume of H_2_ needed to treat widespread fields would simply not be practical from a production/delivery/application, or even from a cost analysis, point of view. The use of H_2_ as a gas may be envisaged on a small scale, such as in some urban settings [66], but again safety considerations would need to be addressed, as would fiscal viability.

To overcome the issue of H_2_ not being usable as a gas, the most common use of hydrogen in treatments is to create a hydrogen-saturated solution, usually by bubbling the water/media with hydrogen gas, using dedicated apparatus that is either commercially available or in development [67]. H_2_ has a limited solubility [43,68], but even so, hydrogen-rich water (HRW) can be easily created. However, from studying the literature it is impossible to predict the exact concentration of H_2_ in such a solution, since a wide range of concentrations are used. HRW can be any solution into which H_2_ is bubbled, so may be saturated (~0.78 mM), supersaturated, or sub-saturated. Therefore, it is very important that any use of HRW is quantified properly. In the biomedical field, a variation of HRW is hydrogen-rich saline (HRS) [69], but this has limited use in plants, especially in agriculture. It might be useful for salt-stressed experiments [70], but it is hard to envisage its widespread use.

In biomedical use, HRW can be orally ingested, but with plants it is added to the soil as feed water or sprayed directly onto foliage [57]. Additional reports demonstrated the positive effects of H_2_ when seeds were treated with HRW, with favorable effects during the germination process [71]. For postharvest storage use, flower stems are placed into HRW [72] or fruits are dipped or sprayed [73]. As discussed below, the use of H_2_-based treatments, such as HRW, may be more useful in floriculture and postharvest storage than in any other aspects of agriculture.

The use of HRW may not be as simple as envisaged. Several studies use HRW in a diluted form. For example, the amounts of 1, 10, 50 and 100% HRW (equivalent to 0.0022 mM, 0.022 mM, 0.11 mM and 0.22 mM H_2,_ respectively) were used in a study of mercury stress alleviation in *Medicago sativa* [74], mitigating oxidative stress and maintaining redox homeostasis. Likewise, 10% HRW (0.022 mM H_2_) was used in a study of cadmium stress, with a particular focus on sulfur metabolism [75]. In a study of aluminum toxicity, 75% HRW (0. 165 mM H_2_) was found to have superior effects, including augmented root development and plant growth, when compared with 100% HRW [76]. A study in cut flowers used a range of concentrations (1%, 10%, 50% and 100%: equivalent to 0.0022 mM, 0.022 mM, 0.11 mM and 0.22 mM H_2,_ respectively) and found that 1% HRW showed the best flower quality, with less onset of senescence [77]. The data available suggest that there may be differential effects on different plant species or plant tissues, and it cannot be assumed that 100% HRW is the best treatment to use. The frequency of the applications may also need to be considered, all adding to the complexity and possible expense, of H_2_-based treatments. Therefore, before the adoption of HRW in any setting, the optimal treatment regime needs to be developed, ensuring that the potential benefits outweigh the costs. To date there has been no wide-scale agricultural use or commercial viability seen in HRW application. 

A further variation of the use of HRW is the application of hydrogen nanobubble water (HNW) [78]. This has been used by others to reduce plant stress, for example, in the alleviation of copper toxicity [79]. Nanobubbles, which have a diameter of less than 500 nm, have a high internal pressure and a large surface area. They also have a negatively charged surface. Together, these properties are purported to aid in the dissolving of H_2_, where the resulting solution is supposedly more stable than HRW, meaning that the H_2_ treatments can be protracted, rather than having a bolus effect. HNW is created by the generation of H_2_-containing water, which is then infused into distilled water using an HNW generator [79]. 

The treatment of biological systems with reactive signaling molecules, such as NO, whether in animals or plants, usually involves the application of a donor molecule [80,81]. However, as well as the inherent toxicity of NO and H_2_S [58,59], donor molecules can also leave behind unwanted and often toxic by-products [82]. Therefore, there are inherit difficulties with these methods which will need to be overcome if they are to be proven effective and adopted in the agri-food industry.

As with the other reactive signaling molecules (e.g., H_2_S, NO etc.), the easy and safe use of molecular hydrogen in biological systems in the future may also involve donor molecules. One emerging compound is AB@hMSN [83], an ammonia, borane-loaded, hollow, mesoporous silica nanoparticle. Such donors may make the storage and transport of H_2_ safer, although, as with other donor molecules used in biological systems, the effects of the donors or their by-products need to be satisfactorily investigated. One such donor molecule is magnesium hydride (MgH_2_)_,_ which gives a longer and more sustained H_2_ release into the solution [84], if used in a special formulation or coating. Even though magnesium is needed by plants, the use of magnesium-based donors would leave magnesium, plus the chemical coating used to passivate the reaction, as by-products. It may also potentially elevate the pH via the production of hydroxide ions with the potential to cause a stress response [85]. The problem of the release of by-products by donors is not restricted to H_2_ treatments. The NO donor, sodium nitroprusside (SNP), was found to release cyanide, and this was also found to have significant effects [86]. This highlights why care must be taken when donor molecules are mooted as an answer to gasotransmitter delivery. The creation of donors will most likely add to the expense of any potential H_2_ applications. The creation of HRW is relatively cheap and easy to perform, so any added expense of using a donor molecule will need to be offset by the added biological benefits (better growth, crop yield or postharvest storage), or allow a cheaper treatment regime, perhaps meaning that the application is less frequent, thus saving material and/or labor costs. The future may also see more analogous compounds being developed and sourced from other industries [87,88] which could reduce the cost of potential donor products.

For H_2_ treatments and applications to be adopted in the UK, strategies for their use need to be put in place. Table 1 summarizes the present options which may be available, once the H_2_ has been produced.

When any H_2_ solution is used, it is important to determine the actual concentration of H_2_ present. There are a variety of methods available to dothis. Some reports use gas chromatography (GC), e.g., [90], whilst others use H_2_ m against a standard calibration curve, e.g., [78], or an assay based on methylene blue [91]. It is only by knowing the actual concentration of the H_2_ present that these studies can be compared. Such details must be known before any pragmatic application regime can be determined and costed. 

## 3. H_2_ and the Alleviation of Plant Stress

Plants, being sessile, are exposed to a range of stress challenges, and this is likely exacerbated by changes in climate, an issue which is predicted to affect the UK [92] and the rest of the world [93]. Improving plant resilience [94], or designing new treatments to mitigate against stress challenges, is important for future food security. The treatments based on molecular hydrogen may be a useful adjunct to other measures in the future. Plants are potentially exposed to a wide range of stresses, including salinity, heavy metals and light, and, since they are sessile, must respond and adapt to these stresses. Although this is an international problem it also applies to the UK, with water quality being a much discussed issue [95]. Therefore, it is important to understand how H_2_ treatments could help to alleviate any of the potential problems the UK agriculture may face in the future.

If H_2_ is to be used in agriculture, there will need to be positive reasons and evidence for its benefits, which outweigh the effort and expense needed for its implementation. As can be seen in Table 2, there is a range of conditions for which H_2_ can be used to alleviate the negative effects of stress in plants.

Plants can suffer from salt stress (NaCl); the connection between climate change and salt stress has been common knowledge for a long time [105]. The salinity levels of arable land are increasing due to irrigation and rising sea levels [106]. Although some treatments, such as the use of selenium [107], have been mooted, the need to engineer plants which are more salt tolerant has been advised [108].

The treatment of plants with H_2_ is found to be beneficial in saline conditions. The pretreatment with HRW was found to increase the germination in rice when seeds were under salt stress [71]. HRW increased germination from 48.87% to 58.36%. There was also a rise in the germination of HRW application with no salt present (85% to 90.47%). HRW was used at 50% and 100% (0.11 mM and 0.22 mM H_2,_ respectively). Before widespread adoption, the question is whether the cost of setting up and executing HRW treatment in such a scenario is less than the cost of the seed saved. As discussed, only large-scale trials and proper cost analysis will answer this, for any seeds used. On a mechanistic level, both HRW treatments were shown to activate α/β-amylase and to increase antioxidant enzymes, particularly superoxide dismutase (SOD), catalase (CAT) and ascorbate peroxidase (APx). Further work in rice, using two cultivars, showed that HRW treatment, either in foliage or through irrigation, increased the dry weight of the plants but decreased plant height, suggesting an increase in plant productivity [70]. However, the increased crop productivity was assumed here, and not measured, so cannot be used to inform any cost/benefit analysis. When the molecular mechanisms involved in the HRW amelioration of salt stress in barley were examined, it was found that the plants had a greater rate of sodium (Na^+^) extrusion, a process mediated by salt overly sensitive (SOS1)-like Na^+^/H^+^ exchange proteins. The plant roots also had a better potassium ion (K^+^) retention, so it was concluded that a better Na^+^/K^+^ ratio accounted for the positive effects caused by HRW treatment [96].

An increasing body of evidence indicates that the treatment with HRW, is beneficial for plants grown in conditions of excess salinity, but clearly there is much more that needs to be established here, including a much wider range of plants used and whether the increased germination or crop yields warrant the use of any H_2_-based application. 

Even though there are environmental regulations in place for industrial pollution in the UK [109], there are still issues where heavy metals accumulate and become a concern [110,111] to agricultural and urban soils [112,113]. Several reports show that H_2_-based treatments may be useful here.

HRW is demonstrated to confer tolerance to mercury in alfalfa (*M. sativa* L. cv. Biaogan) seedlings [74]. Alfalfa is a valuable forage crop, so this would complicate any cost/benefit analysis. However, 10% HRW (0.022 mM) led to relief of the effect of mercury, showing a reduction of approximately 50% of the fresh weight and dry weight of the roots seen in the mercury treatment. This equated to an approximate increase of 10% in growth with the use of HRW when mercury was present. Significantly, the authors state that alfalfa is often grown in areas where heavy metal content is high, and so HRW use in such situations may be worthwhile. On a cellular level, the activities of hydrogen peroxide (H_2_O_2_)-removing enzymes were increased and the intracellular glutathione ratios (GSH:GSSG) were restored by HRW. The overall oxidative stress, induced by mercury, was also reduced and the plants were less stunted. Cadmium (Cd) ion stress was also reduced by HRW in Medicago [97]. Ten percent HRW (0.022 mM) led to an increase of 20% of the fresh weight of root material. No effect of 10% HRW was seen in plants which was not stressed with the heavy metals. However, in HRW-treated plants, Cd uptake was inhibited, and again, as with mercury stress, HRW reduced oxidative stress and the cellular redox status was maintained. Similar data were reported for Chinese cabbage (*Brassica campestris* spp. *Chinensis* L.), where HRW increased Cd tolerance, whilst also favorably altering the antioxidant capacity of the plants’ cells [98]. The fresh weight of the plants was increased by 16% with the treatment of 50% HRW (0.11 mM), with root elongation improving by 48%. More recent data show that the movement of calcium ions (Ca^2+^) across the plasma membrane and the generation of H_2_O_2_ were important in mediating the effects of HRW [99]. Further work in *Brassica chinensis* (Dongfang 2) (Pak choi) and *Arabidopsis thaliana* showed that the transporter proteins, iron-regulated transporter 1 (IRT1) and zinc-regulated transporter protein 2 (ZIP2), were important for mediating HRW actions [100]. Here, 50% HRW (0.11 mM) relieved the effects of 1 µM and 5 µM Cd on the fresh weight of Pak choi roots by about 50%, but there was no effect of Cd toxicity on the overall plant growth, so HRW could not be deemed beneficial in this instance. This would cast into doubt the benefits gained by the expense of the HRW treatment of crops under this particular level of heavy-metal challenge.

Although not classed as a heavy metal, aluminum (Al) ions are toxic to plants [114] and their presence is regularly monitored in the environment in the UK [115,116]. As has been reported in heavy metals, the treatment with HRW can alleviate the stress induced by the presence of this aluminum ions [101]. The uptake of aluminum ions was reduced by HRW treatment, which alleviated the growth inhibition that the aluminum treatment induced. Interestingly, the data showed that the effects of HRW may be mediated by a reduction in nitric oxide (NO) accumulation in the cells. However, even though a pre-treatment with 50% (0.11 mM) HRW partly alleviated the effects of Al on roots, and 10% (0.022 mM) HRW caused an increase in root elongation without any Al stress. when the HRW and Al were added together there seemed to be no effect of the presence of H_2_. This casts doubt over the use of this treatment in the field, where the stress is present before H_2_ can be used as a treatment. 

H_2_, administered as HRW, has, therefore, been found to bestow metal ion tolerance to several plant species, and the mechanisms of action are starting to be discovered. However, the use of HRW for agricultural purposes needs far more research before it can be suggested as commercially viable.

As with other areas of the world [117], the UK temperatures are projected to increase due to climate change [118,119]. The recent Intergovernmental Panel on Climate Change (IPCC) report [120] shows that global temperature rises are inevitable and need to be taken seriously. Therefore, agricultural practices need to help mitigate this for sustained food security, as well as the other stresses. Once again, H_2_-based treatments may be of use. In a study of heat stress in cucumber, seedlings were treated with 50 or 100% HRW (0.11 mM and 0.22 mM H_2,_ respectively) for seven days, and then subjected to three days of heat. Leaf curling caused by the heat was reduced, but there was no effect on root growth. Therefore, the commercial value of the crop may be increased, but not the overall yield. In line with the other commercially important crops mentioned, the antioxidant capacity was increased in these plants, along with an increased expression of the heat shock protein 70 (HSP70). Therefore, the authors concluded that HRW increased the plants’ tolerance to heat [102]. So, as with other plant stress exposures, HRW, or future H_2_ treatments, may be useful in UK agriculture by supporting plant responses and enhancing tolerance to rising and fluctuating temperatures [120]. 

As the climate and atmospheric ozone levels change, the light to which plants are exposed may alter, perhaps through changes in UV irradiation [121,122]. Clearly, the appropriate light intensity and quality is vital for optimal plant growth and crop production. 

The exposure of Medicago to UV-B radiation increased endogenous H_2_ production, an action that was mimicked by the treatment with HRW. The effects of H_2_ appeared to be mediated by alterations in the cellular antioxidant capacity, partly through the modulation of flavonoid levels [104]. Twenty-two types of flavonoids were shown to have their levels altered by HRW [104]. In a study exposing radish (*Raphanus sativus* L.) to UV-A it was found that the effects were mediated by increases in anthocyanin. Furthermore, it was suggested that plant hormones, mitogen-activated protein kinases (MAPKs) and Ca^2+^ were involved in this mechanism [103]. However, there were no quantification data on the size of the plants, and from the photograph shown there was little effect of HRW on plant size.

It is becoming clear that there are a range of plant stresses for which the use of H_2_-based treatments may be useful (Table 2). Many of these are relevant to the UK, and many of these stress challenges are only likely to increase as the climate of the UK changes and pernicious, anthropogenic activity continues [120]. However, the evidence for increased growth and crop production is not exhaustive and much more needs to be investigated before a persuasive argument to adopt H_2_-based treatments in the UK can be made. On the other hand, it is not just plant/crop growth which may be influenced by H_2_ treatment, but the subsequent storage and transportation of produce may also benefit from H_2_-based treatments.

## 4. Postharvest Senescence and the Beneficial Effects of H_2_

Crops can be treated with H_2_ postharvest, and this may be one of the most effective ways that H_2_ could be used in future UK industries. It was estimated that, in the UK, the food supply chain accounts for 13.1 Mt (13,107,000 tones) of food waste, with around 60% of this waste being comprised of cereals and vegetables [123]. Therefore, any improvements to the transport and storage of crops would have a large impact on future food security. To date H_2_ is shown to be beneficial in several relevant scenarios (Table 3).

As considered above, one of the easiest ways to use H_2_-based treatments is to create HRW. This can be sprayed onto the surface of fruits and vegetables or, alternatively, the produce can be dipped into HRW. For the floriculture industry, the flower stems can simply be placed directly into the HRW. As HRW is relatively cheap, easy to use, leaves no harmful by-products, and is safe for human health, the use of such treatments in postharvest may have the most potential for future H_2_ adoption in the UK (Table 3).

A range of HRW concentrations has also been used to treat kiwifruit, with 80% (0.176 mM H_2_) giving the best results: that is, the fruit had less rot and a better firmness when compared with the other treatments [73]. This benefit was seen over a period of two weeks, which could increase the commercial value of the crop. The lipid peroxidation was reduced, whilst the activity of the endogenous antioxidant, SOD, was increased [60]. H_2_ fumigation also decreased kiwifruit senescence, and this appeared to involve alterations in ethylene metabolism [124]. Here, the results showed that the firmness of fruit was increased, with the ripening process being slowed, especially when using a two-stage treatment with 4.5 μL L^−1^ H_2_. Interestingly, the damage caused by the pathogen, *Phomopsis*, was also decreased by H_2_. This suggests that the storage and transport of such fruit may incur reduced losses if H_2_ is used. More recently, H_2_ was shown to increase the storage time of Chinese chive (*Allium tuberosum* Rottler ex Spreng) [125] by decreasing oxidative stress in the tissues. Significant positive changes were seen in weight loss, decay index and soluble protein content on H_2_ treatment after 4 days, again suggesting that the storage time, and therefore commercial value, of this product would be increased with the application of H_2_. Similar data were reported for the fungus, *Hypsizygus marmoreus*, where 25% HRW resulted in approximately 25% less weight loss and a higher firmness of the mushrooms. Again, the modulation of antioxidants and the reduction in oxidative stress seemed to be the driving factors behind the efficacious outcomes reported [126]. Other studies in *Hypsizygus marmoreus* showed that H_2_ at 0.8 mM could mitigate against several stresses, including CdCl_2_, NaCl and H_2_O_2_. Again, antioxidants and the lowering of oxidative stress seemed to be important [129], although this study was not carried out postharvest. However, the lower concentrations of H_2_ in HRW (0.195 mM and 0.585 mM H_2_) did reduce senescence in postharvest tomato [90], with the firmness being significantly improved for up to 16 days in storage. The nitrite accumulation was also reduced in these fruits. By looking at the relevant enzymes involved in nitrite metabolism, it was found that the enzyme responsible for nitrite generation, i.e., nitrate reductase (NR), was decreased, whilst the enzyme used for nitrite removal to ammonia, i.e., nitrite reductase (NiR), was increased. Vitamin C was also maintained in the tomatoes, and the authors suggested that H_2_ would be beneficial for the commercial storage of this fruit. Furthermore, it was suggested that H_2_ could be used for a range of crop storage, with the data showing positive effects in lettuce, eggplant, spinach, apple and carrots, as well as daylily flowers [90]. 

Several studies showed that H_2_ treatment was beneficial for cut flowers (Table 3). In cut lily and rose stems, HRW increased the vase life and flowerhead diameters. The vase life of lilies increased by approximately 2.5 days, whilst for roses HRW led to an increased vase life of 3 days. This could be significant for the commercial value of such flowers. On a cellular level, the stomata size was reduced and the effects were mediated by reduced oxidative stress [72]. Further studies in cut lilies, investigating the crosstalk between H_2_ and NO, combined with proteomic analysis, revealed that the effects of increased vase life may be mediated by the expression of the chloroplastic protein ATP synthase CF1 alpha subunit (AtpA) [127].

Treatment with H_2_ increased the vase quality of cut lisianthus, an effect that was mediated by the increases in autogenous antioxidants and the subsequent reduction in oxidative stress, thereby maintaining the cellular redox and allowing the flowers to last longer [128]. The treatment with very low H_2_ concentrations (0.078 mM H_2_) increased the vase life of the flowers by 4 days. In addition, ethylene metabolism was implicated in the increased vase life of cut roses [77], where the maximum effect of H_2_ (1% HRW (0.0047 mg L^−1^) was reported to be a little over 2 days. In cut carnations, an H_2_ donor (magnesium hydride) increased the vase life by approximately 2 days and further investigation showed that the gas, H_2_S, was found to be involved in mediating this response [84].

H_2_ treatment postharvest may not be the only option. In a study of daylily [130], the preharvest application of HRW improved the crop yield (by approximately 10%) and also the flower quality, especially in the chilling stress, which may be useful when considering postharvest transportation and storage. 

What is clear from such a collection of data is that treatments with H_2_, whether as a gas in solution or from donor molecules, can increase the quality of postharvest plant produce. This was shown for a range of fruits and flowers in which many H_2_-based treatments were used. Therefore, more work needs to be carried out to optimize the delivery of H_2_ and ensure that it is tailored to the crop being used. This safe and easy-to-use manipulation of postharvest plant tissues has great promise for the future of the food and horticultural industries within the UK and internationally. However, as discussed above, the adoption of any H_2_-based treatment comes down to a cost/benefit analysis. Several fruits appear to ripen more slowly and retain their firmness, but, unless the amount and value of the sellable product outweighs the cost of treatment, it may be more commercially effective to simply discard the unwanted crop (or use it for compost or fuel), rather than attempt to prolong its commercial viability. On the flipside of this is whether the crop is plentiful enough to allow such waste. As food security becomes a bigger issue around the globe, all treatments which mitigate food loss may become important, and H_2_-based treatments may be a useful adjunct to other applications.

## 5. Conclusions and the Future

Climate change will continue to be an issue for the UK [92], as well as globally [93,120], and agriculture will be impacted by a range of stress challenges, including those caused by anthropogenic activities [131]. 

As a mitigating strategy, the use of H_2_ as an agricultural treatment may have a future [1,2], although much more evidence needs to be gathered before a good case for its widespread adoption in agricultural practices can be made. The use of H_2_ gas may not be easy or pragmatic; however, HRW can be used to encourage healthy germination, as a foliar or as root treatment, although the optimal concentration of HRW needs to be determined for different plant species and applications. An alternate approach is to use donor molecules, which can release H_2_ over a longer timeframe, although these must be deemed safe if used on food products. However, any commercial treatment has to be beneficial and a deficiency in crops may be more favorable than the use of costly applications. Large-scale H_2_ use relies on other industries, but it is unlikely that the field agricultural practice in the UK will adopt H_2_ in the foreseeable future without the necessary agricultural trials.

Although it is clear that H_2_ can be used for the alleviation of a range of stresses, including salinity, heavy metals, temperature and light stresses (Table 2), potentially the most promising use of H_2_ treatment is in allaying postharvest senescence (Table 3). As with food production around the globe, there are significant losses in the UK [123]; mitigating these losses would be of tangible value, not only to the economy, but to the food security for the country’s population. The treatment of fruit and flowers with HRW is far easier than spraying a whole field, and will no doubt be seen to be commercially more viable as an option for the use of H_2_-based technologies. It is suggested here that, because HRW is relatively cheap to produce and is easy to use, its adoption in postharvest should be a priority. The effects of HRW at postharvest are evident if plants are treated at preharvest [124]. However, a clear cost analysis would still need to be carried out by those prepared to adopt H_2_ applications [132]. As of yet, little data are available on any agricultural cost analysis. It is hard to translate a reported increase in root length or wet weight, into the subsequent consequences of this for crop yield, especially if it is a foraging crop [74]. Therefore, large-scale trials with all the costs and cost benefits measured for a range of important crops are necessary. Such research would need to be carried out in different regions of the world and under different conditions. Most of the positive effects of H_2_ are seen when plants are stressed, and there may be little or no effect if plants are grown under optimal conditions, maintained by other cheaper treatments. However, as H_2_ is adopted by other industries [133] it is likely that its generation, storage and transportation will become cheaper, making it more attractive in an agricultural setting. H_2_ treatments, as HRW, may be combined with other treatments, such as fertilizer, also reducing the dedicated application costs. Even if the current costs are prohibitive, the use of H_2_-based treatments are likely to be pragmatic in the future, and this may be especially useful for a range of crops at postharvest.

The exact mechanisms of the cellular actions of H_2_ in plants, or indeed animals, are still not clear. Despite this dearth of clarity on the direct action of H_2_, it is apparent that H_2_-based treatments are useful to ameliorate stress responses in plants; therefore, the use of molecular hydrogen to enhance agricultural and, particularly, floricultural and horticultural outputs should be considered for wider use in the UK. This may be most important for decreasing food waste and increasing food security.

## Figures and Tables

**Table 1:** A summary of treatments which may be of potential use in future UK agriculture.

Treatment	Comments	References
H_2_ as a gas	Safety issues, such as flammability and gas being lighter than air, make it hard to administer. Likely not easy to adopt widely. Useful in biomedical arenas.	[65,89]
Hydrogen-rich water (HRW)	Likely the preferred method for current plant treatment, either soil feedwater or foliar. Requires optimization for any plant being treated. May require repeated treatments, so adds to expense.	[70,72]
Hydrogen-rich saline (HRS)	Not useful for agricultural use. Mainly useful for biomedical use, unless considering plant salt stress.	[69]
Hydrogen nanobubble water (HNW)	Has better H_2_ solubility and sustained H_2_ release. May require more specialist equipment and expense, although may decrease the regularly of applications needed.	[78]
Magnesium hydride (MgH_2_)	Potentially provides longer kinetics of action. May leave behind unwanted by-products.	[84]
AB@hMSN	Long H_2_ release. Leaves behind by-products for which the effects need to be determined. Increases the expense of application but might reduce treatment frequencies.	[83]
Other hydrogen donors	Needs future investment and is cognizant of by-products. ay be an increase in expense, unless widely adopted by other industries.	[87,88]

**Table 2:** A selection of plant stresses and the effects of molecular hydrogen.

Stress	Comments/Effects Seen on H_2_ Treatment	References
Salt	Salt stress is important in coastal regions, especially if there is sea ingress because of climate change. Stress ameliorated; antioxidants increased; Na^+^ and K^+^ movements altered.	[70,71,96]
Mercury	Reduced oxidative stress; redox status maintained; better growth.	[74]
Cadmium (Cd)	Cd uptake reduced; oxidative stress lowered; redox maintained. Ca^2+^, H_2_O_2_, iron-regulated transporter 1 (*IRT1*) and zinc-regulated transporter protein 2 *(ZIP2*) are all implicated.	[72,97,98,99,100]
Aluminum (Al)	Al uptake reduced. Nitric oxide (NO) accumulation lowered.	[101]
Extreme temperature	Climate change may lead to more extreme temperatures, both high and low, depending on the geographical region. Increased antioxidants, photosynthetic capacity and heat shock protein 70 (HSP70) expression.	[102]
UV-A light	Increased levels of anthocyanin.	[103]
UV-B light	Alterations of antioxidants and flavonoids.	[104]

**Table 3:** Examples of the use of H_2_ treatments for postharvest of plant materials. Flowers are all cut. H_2_ concentrations are calculated from information given in relevant papers.

Plant Material Being Treated	Treatment	Effects Seen	Reference
Kiwifruit	HRW (80% best: 0.176 mM H_2_)	Increased firmness and less rot. Lower oxidative stress.	[73]
Kiwifruit	H_2_ fumigation	Delayed senescence. Involves ethylene metabolism.	[124]
Chinese chive	1%, 2% or 3% H_2_ fumigation	Reduced oxidative stress.	[125]
*Hypsizygus marmoreus*	HRW (25% best: 0.2 mM H_2_)	Reduced oxidative stress.	[126]
Tomato (*Solanum lycopersicum* cv.)	HRW (0.195 mM and 0.585 mM H_2_)	Delayed senescence and less nitrite accumulation. Vitamin C retained.	[90]
Flowers of lily and rose (*Lilium* spp. & *Rosa hybrid* L.)	HRW (0.5% 2.25 µM H_2_) and 1% (4.5 µM H_2_); lily: 50% (0.225 mM H_2_); rose	Improved vase life. Greater flower diameter. Less oxidative stress.	[72]
Lily flowers (*Lilium* “Manissa”)	HRW (1%: 0.0022 mM H_2_) and 150 μM sodium nitroprusside (SNP)	Enhanced flower freshness. ATP synthase CF1 alpha subunit (AtpA) up-regulated.	[127]
Lisianthus flowers	HRW (0.078 mM H_2_)	Vase life extended. Redox maintained as less oxidative stress.	[128]
Rose ‘Movie star’ flowers	HRW (1% best: 0.00235 mM H_2_)	Less flower senescence, mediated by ethylene metabolism.	[77]
Carnation flowers	MgH_2_ (0.1 g L^–1^) with citrate	Flower quality improved. H_2_S signaling downstream of H_2_.	[84]
Carnation flowers (*Dianthuscaryophyllus* L.).	Hydrogen nanobubble water (HNW): 5% best (0.025 mM H_2_)	Less senescence so longer vase life. Lower oxidative stress.	[78]

## Data Availability

No new data was reported here.

## References

[1] Zeng J., Ye Z., Sun X. (2014). Progress in the study of biological effects of hydrogen on higher plants and its promising application in agriculture. Med. Gas Res..

[2] Li C., Gong T., Bian B., Liao W. (2018). Roles of hydrogen gas in plants: A review. Funct. Plant Biol..

[3] Ohta S. (2011). Recent progress toward hydrogen medicine: Potential of molecular hydrogen for preventive and therapeutic applications. Curr. Pharm. Des..

[4] Ge L., Yang M., Yang N.-N., Yin X.-X., Song W.-G. (2017). Molecular hydrogen: A preventive and therapeutic medical gas for various diseases. Oncotarget.

[5] Alwazeer D., Liu F.F.-C., Wu X.Y., LeBaron T.W. (2021). Combating oxidative stress and inflammation in COVID-19 by molecular hydrogen therapy: Mechanisms and perspectives. Oxidative Med. Cell. Longev..

[6] Chen K., Lin W., Kuo H. (2021). Chemical and biochemical aspects of molecular hydrogen in treating Kawasaki Disease and COVID-19. Chem. Res. Toxicol..

[7] Russell G., Nenov A., Hancock J. (2021). Oxy-hydrogen gas: The rationale behind its use as a novel and sustainable treatment for COVID-19 and other respiratory diseases. Eur. Med. J..

[8] Guan W., Wei C., Chen A., Sun X., Guo G., Zou X., Shi J., Lai P., Zheng Z., Zhong N. (2020). Hydrogen/oxygen mixed gas inhalation improves disease severity and dyspnea in patients with Coronavirus disease 2019 in a recent multicentre, open-label clinical trial. J. Thorac. Dis..

[9] Ohno K., Ito M., Ichihara M., Ito M. (2012). Molecular hydrogen as an emerging therapeutic medical gas for neurodegenerative and other diseases. Oxidative Med. Cell. Longev..

[10] Chen W., Zhang H., Qin S. (2021). Neuroprotective effects of molecular hydrogen: A critical review. Neurosci. Bull..

[11] Yoritaka A., Takanashi M., Hirayama M., Nakahara T., Ohta S., Hattori N. (2013). Pilot study of H_2_ therapy in Parkinson’s Disease: A randomized double-blind placebo-controlled trial. Mov. Disord..

[12] Ishibashi T., Sato B., Shibata S., Sakai T., Naritomi Y., Koyanagi S., Hara H., Nagao T. (2014). Therapeutic efficacy of infused molecular hydrogen in saline on rheumatoid arthritis: A randomized, double-blind, placebo-controlled pilot study. Int. Immunopharmacol..

[13] Kajiyama S., Hasegawa G., Asano M., Hosoda H., Fukui M., Nakamura N., Kitawaki J., Imai S., Nakano K., Ohta M. (2008). Supplementation of hydrogen-rich water improves lipid and glucose metabolism in patients with type 2 diabetes or impaired glucose tolerance. Nutr. Res..

[14] LeBaron T.W., Singh R.B., Fatima G., Kartikey K., Sharma J.P., Ostojic S.M., Gvozdjakova A., Kura B., Noda M., Mojto V. (2020). The effects of 24-week, high-concentration Hydrogen-Rich Water on body composition, blood lipid profiles and inflammation biomarkers in men and women with Metabolic Syndrome: A randomized controlled trial. Diabetes Metab. Syndr. Obes..

[15] Xia C., Liu W., Zeng D., Zhu L., Sun X., Sun X. (2013). Effect of hydrogen-rich water on oxidative stress, liver function, and viral load in patients with chronic Hepatitis B. Clin. Transl. Sci..

[16] Kang K., Kang Y., Choi I., Gu Y., Kawamura T., Toyoda Y., Nakao A. (2011). Effects of drinking hydrogen-rich water on the quality of life of patients treated with radiotherapy for liver tumors. Med. Gas Res..

[17] Li L., Lou W., Kong L., Shen W. (2021). Hydrogen commonly applicable from medicine to agriculture: From molecular mechanisms to the field. Curr. Pharm. Des..

[18] Russell G., Zulfiqar F., Hancock J.T. (2020). Hydrogenases and the role of molecular hydrogen in plants. Plants.

[19] Vargas S.R., Santos P.V.D., Giraldi L.A., Zaiat M., Calijuri M.D.C. (2018). Anaerobic phototrophic processes of hydrogen production by different strains of microalgae *Chlamydomonas* sp. FEMS Microbiol. Lett..

[20] Scranton M.A., Ostrand J.T., Fields F.J., Mayfield S.P. (2015). *Chlamydomonas* as a model for biofuels and bio-products production. Plant J..

[21] Ben-Zvi O., Dafni E., Feldman Y., Yacoby I. (2019). Re-routing photosynthetic energy for continuous hydrogen production in vivo. Biotechnol. Biofuels.

[22] Piché-Choquette S., Constant P. (2019). Molecular hydrogen, a neglected key driver of soil biogeochemical processes. Appl. Environ. Microbiol..

[23] Debbarma J., Sarki Y.N., Saikia B., Boruah H.P.D., Singha D.L., Chikkaputtaiah C. (2019). Ethylene response factor (ERF) family proteins in abiotic stresses and CRISPR–Cas9 genome editing of ERFs for multiple abiotic stress tolerance in crop plants: A review. Mol. Biotechnol..

[24] Kolbert Z., Feigl G., Freschi L., Poór P. (2019). Gasotransmitters in action: Nitric oxide-ethylene crosstalk during plant growth and abiotic stress responses. Antioxidants.

[25] Pandey A.K., Gautam A. (2020). Stress responsive gene regulation in relation to hydrogen sulfide in plants under abiotic stress. Physiol. Plant..

[26] Corpas F.J., González-Gordo S., Cañas A., Palma J.M. (2019). Nitric oxide and hydrogen sulfide in plants: Which comes first?. J. Exp. Bot..

[27] Kolbert Z., Barroso J.B., Brouquisse R., Corpas F.J., Gupta K.J., Lindermayr C., Loake G.J., Palma J.M., Petřivalský M., Wendehenne D. (2019). A forty year journey: The generation and roles of NO in plants. Nitric Oxide.

[28] Lisjak M., Teklic T., Wilson I.D., Whiteman M., Hancock J.T. (2013). Hydrogen sulfide: Environmental factor or signalling molecule?. Plant Cell Environ..

[29] Wilson H.R., Veal D., Whiteman M., Hancock J.T. (2017). Hydrogen gas and its role in cell signalling. CAB Rev..

[30] Lubega J., Umbreen S., Loake G.J. (2021). Recent advances in the regulation of plant immunity by *S*-nitrosylation. J. Exp. Bot..

[31] Aroca A., Gotor C., Romero L.C. (2018). Hydrogen sulfide signaling in plants: Emerging roles of protein persulfidation. Front. Plant Sci..

[32] Kaya C., Ashraf M., Akram N.A. (2018). Hydrogen sulfide regulates the levels of key metabolites and antioxidant defense system to counteract oxidative stress in pepper (*Capsicum annuum* L.) plants exposed to high zinc regime. Environ. Sci. Pollut. Res..

[33] Souri Z., Karimi N., Farooq M.A., Sandalio L.M. (2020). Nitric oxide improves tolerance to arsenic stress in *Isatis cappadocica* desv. Shoots by enhancing antioxidant defenses. Chemosphere.

[34] Hancock J.T., Whiteman M. (2014). Hydrogen sulfide and cell signaling: Team player or referee?. Plant Physiol. Biochem..

[35] Hancock J.T., Whiteman M. (2016). Hydrogen sulfide signaling: Interactions with nitric oxide and reactive oxygen species. Ann. New York Acad. Sci..

[36] Hancock J.T., Neill S.J. (2019). Nitric oxide: Its generation and interactions with other reactive signaling compounds. Plants.

[37] Bhuyan M.B., Hasanuzzaman M., Parvin K., Mohsin S.M., Al Mahmud J., Nahar K., Fujita M. (2020). Nitric oxide and hydrogen sulfide: Two intimate collaborators regulating plant defense against abiotic stress. Plant Growth Regul..

[38] Liu F., Jiang W., Han W., Li J., Liu Y. (2017). Effects of hydrogen-rich water on fitness parameters of rice plants. Agron. J..

[39] Zeng J., Zhang M., Sun X. (2013). Molecular hydrogen is involved in phytohormone signaling and stress responses in plants. PLoS ONE.

[40] Wu Q., Su N., Huang X., Ling X., Yu M., Cui J., Shabala S. (2020). Hydrogen-rich water promotes elongation of hypocotyls and roots in plants through mediating the level of endogenous gibberellin and auxin. Funct. Plant Biol..

[41] Nakao A., Toyoda Y., Sharma P., Evans M., Guthrie N. (2010). Effectiveness of hydrogen rich water on antioxidant status of subjects with potential metabolic syndrome—an open label pilot study. J. Clin. Biochem. Nutr..

[42] Zhang J.Y., Song S.D., Pang Q., Zhang R.Y., Wan Y., Yuan D.W., Wu Q.F., Liu C. (2015). Hydrogen-rich water protects against acetaminophen-induced hepatotoxicity in mice. World J. Gastroenterol..

[43] Hancock J.T., Russell G. (2021). Downstream signalling from molecular hydrogen. Plants.

[44] Chouchani E.T., James A.M., Fearnley I.M., Lilley K.S., Murphy M.P. (2011). Proteomic approaches to the characterization of protein thiol modification. Curr. Opin. Chem. Biol..

[45] Ohsawa I., Ishikawa M., Takahashi K., Watanabe M., Nishimaki K., Yamagata K., Katsura K., Katayama Y., Asoh S., Ohta S. (2007). Hydrogen acts as a therapeutic antioxidant by selectively reducing cytotoxic oxygen radicals. Nat. Med..

[46] Penders J., Kissner R., Koppenol W.H. (2014). ONOOH does not react with H_2_: Potential beneficial effects of H_2_ as an antioxidant by selective reaction with hydroxyl radicals and peroxynitrite. Free Radic. Biol. Med..

[47] LeBaron T.W., Kura B., Kalocayova B., Tribulova N., Slezak J. (2019). A new approach for the prevention and treatment of cardiovascular disorders. Molecular hydrogen significantly reduces the effects of oxidative stress. Molecules.

[48] Hancock J.T., LeBaron T.W., Russell G. (2021). Molecular hydrogen: Redox reactions and possible biological interactions. React. Oxyg. Species.

[49] Hancock J.T., Hancock T.H. (2018). Hydrogen gas, ROS metabolism and cell signaling: Are hydrogen spin states important. React. Oxyg. Species.

[50] Chen H.G., Xie K.L., Han H.Z., Wang W.N., Liu D.Q., Wang G.L., Yu Y.H. (2013). Heme oxygenase-1 mediates the anti-inflammatory effect of molecular hydrogen in LPS-stimulated RAW 264.7 macrophages. Int. J. Surg..

[51] Li S., Fujino M., Takahara T., Li X.K. (2019). Protective role of heme oxygenase-1 in fatty liver ischemia–reperfusion injury. Med. Mol. Morphol..

[52] Jin Q., Zhu K., Cui W., Xie Y., Han B.I.N., Shen W. (2013). Hydrogen gas acts as a novel bioactive molecule in enhancing plant tolerance to paraquat-induced oxidative stress via the modulation of heme oxygenase-1 signalling system. Plant Cell Environ..

[53] Lin Y., Zhang W., Qi F., Cui W., Xie Y., Shen W. (2014). Hydrogen-rich water regulates cucumber adventitious root development in a heme oxygenase-1/carbon monoxide-dependent manner. J. Plant Physiol..

[54] Ren A., Liu R., Miao Z.G., Zhang X., Cao P.F., Chen T.X., Li C.Y., Shi L., Jiang A.L., Zhao M.W. (2017). Hydrogen-rich water regulates effects of ROS balance on morphology, growth and secondary metabolism via glutathione peroxidase in *Ganoderma lucidum*. Environ. Microbiol..

[55] Marvasi M. (2017). Potential use and perspectives of nitric oxide donors in agriculture. J. Sci. Food Agric..

[56] Dooley F.D., Nair S.P., Ward P.D. (2013). Increased growth and germination success in plants following hydrogen sulfide administration. PLoS ONE.

[57] Zhu L., Wang W., Shi J., Zhang W., Shen Y., Du H., Wu S. (2014). Hydrogen sulfide extends the postharvest life and enhances antioxidant activity of kiwifruit during storage. J. Sci. Food Agric..

[58] Weinberger B.l., Laskin D.L., Heck D.E., Laskin J.D. (2001). The toxicology of inhaled nitric oxide. Toxicol. Sci..

[59] Truong D.H., Eghbal M.A., Hindmarsh W., Roth S.H., O'Brien P.J. (2006). Molecular mechanisms of hydrogen sulfide toxicity. Drug Metab. Rev..

[60] Safety of Inhaled Hydrogen Gas Mixtures in Healthy Volunteers. https://clinicaltrials.gov/ct2/show/NCT04046211?term=molecular+hydrogen&draw=3&rank=14.

[61] Crowl D.A., Jo Y.D. (2007). The hazards and risks of hydrogen. J. Loss Prev. Process Ind..

[62] Jin T., Liu Y., Wei J., Wu M., Lei G., Chen H., Yuqi Lan Y. (2017). Modeling and analysis of the flammable vapor cloud formed by liquid hydrogen spills. Int. J. Hydrog. Energy.

[63] McCarthy S.D., González H.E., Higgins B.D. (2020). Future trends in nebulized therapies for pulmonary disease. J. Pers. Med..

[64] Brewer G.D. (2017). Hydrogen Aircraft Technology.

[65] Huang C.S., Kawamura T., Toyoda Y., Nakao A. (2010). Recent advances in hydrogen research as a therapeutic medical gas. Free Radic. Res..

[66] Edmondson J.L., Cunningham H., Tingley D.O.D., Dobson M.C., Grafius D.R., Leake J.R., McHugh N., Nickles J., Phoenix G.K., Ryan A.J. (2020). The hidden potential of urban horticulture. Nat. Food.

[67] Li Q., Asada R., Tanaka Y., Miwa N. (2017). Fundamental insight into the methodology of hydrogen water in biological studies. J. Nanosci. Nanotechnol..

[68] Wilhelm E., Battino R., Wilcock R.J. (1977). Low-pressure solubility of gases in liquid water. Chem. Rev..

[69] Sun H., Chen L., Zhou W., Hu L., Li L., Tu Q., Chang Y., Liu Q., Sun X., Wu M. (2011). The protective role of hydrogen-rich saline in experimental liver injury in mice. J. Hepatol..

[70] Fu X., Ma L., Gui R., Li Y., Yang X., Zhang J., Imran M., Tang X., Tian H., Mo Z. (2020). Hydrogen rich water (HRW) induces plant growth and physiological attributes in fragrant rice varieties under salt stress. Res. Sq..

[71] Xu S., Zhu S., Jiang Y., Wang N., Wang R., Shen W., Yang J. (2013). Hydrogen-rich water alleviates salt stress in rice during seed germination. Plant Soil.

[72] Ren P.J., Jin X., Liao W.B., Wang M., Niu L.J., Li X.P., Xu X.T., Zhu Y.C. (2017). Effect of hydrogen-rich water on vase life and quality in cut lily and rose flowers. Hortic. Environ. Biotechnol..

[73] Hu H., Li P., Wang Y., Gu R. (2014). Hydrogen-rich water delays postharvest ripening and senescence of kiwifruit. Food Chem..

[74] Cui W., Fang P., Zhu K., Mao Y., Gao C., Xie Y., Wang J., Shen W. (2014). Hydrogen-rich water confers plant tolerance to mercury toxicity in alfalfa seedlings. Ecotoxicol. Environ. Saf..

[75] Cui W., Yao P., Pan J., Dai C., Cao H., Chen Z., Zhang S., Xu S., Shen W. (2020). Transcriptome analysis reveals insight into molecular hydrogen-induced cadmium tolerance in alfalfa: The prominent role of sulfur and (homo) glutathione metabolism. BMC Plant Biol..

[76] Zhao X., Chen Q., Wang Y., Shen Z., Shen W., Xu X. (2017). Hydrogen-rich water induces aluminum tolerance in maize seedlings by enhancing antioxidant capacities and nutrient homeostasis. Ecotoxicol. Environ. Saf..

[77] Wang C., Fang H., Gong T., Zhang J., Niu L., Huang D., Huo J., Liao W. (2020). Hydrogen gas alleviates postharvest senescence of cut rose ‘Movie star’by antagonizing ethylene. Plant Mol. Biol..

[78] Li L., Yin Q., Zhang T., Cheng P., Xu S., Shen W. (2021). 2021. Hydrogen nanobubble water delays petal senescence and prolongs the vase life of cut carnation (*Dianthus caryophyllus* L.) flowers. Plants.

[79] Fan W., Zhang Y., Liu S., Li X., Li J. (2020). Alleviation of copper toxicity in *Daphnia magna* by hydrogen nanobubble water. J. Hazard. Mater..

[80] Song Z.J., Ng M.Y., Lee Z.W., Dai W., Hagen T., Moore P.K., Huang D., Deng L.W., Tan C.H. (2014). Hydrogen sulfide donors in research and drug development. Med. Chem. Comm..

[81] Ederli L., Reale L., Madeo L., Ferranti F., Gehring C., Fornaciari M., Romano B., Pasqualini S. (2009). NO release by nitric oxide donors in vitro and *in planta*. Plant Physiol. Biochem..

[82] Bethke P.C., Libourel I.G., Reinöhl V., Jones R.L. (2006). Sodium nitroprusside, cyanide, nitrite, and nitrate break Arabidopsis seed dormancy in a nitric oxide-dependent manner. Planta.

[83] Wang Y., Lv P., Kong L., Shen W., He Q. (2021). Nanomaterial-mediated sustainable hydrogen supply induces lateral root formation via nitrate reductase-dependent nitric oxide. Chem. Eng. J..

[84] Li L., Liu Y., Wang S., Zou J., Ding W., Shen W. (2020). Magnesium hydride-mediated sustainable hydrogen supply prolongs the vase life of cut carnation flowers via hydrogen sulfide. Front. Plant Sci..

[85] Guo W., Chen S., Hussain N., Cong Y., Liang Z., Chen K. (2015). Magnesium stress signaling in plant: Just a beginning. Plant Signal. Behav..

[86] Bethke P.C., Libourel I.G., Jones R.L. (2006). Nitric oxide reduces seed dormancy in Arabidopsis. J. Exp. Bot..

[87] Alemán-Vázquez L.O., Torres-Mancera P., Ancheyta J., Ramírez-Salgado J. (2016). Use of hydrogen donors for partial upgrading of heavy petroleum. Energy Fuels.

[88] Mayorga M.A., Cadavid J., Lopez C., Suarez O., Bonilla J., Vargas J., Narvaez P., Rodriguez L.I. (2020). Selection of hydrogen donors for the production of renewable diesel by in situ catalytic deoxygenation of palm oil. Chem. Eng. Trans..

[89] Slezak J., Kura B., LeBaron T.W., Singal P.K., Buday J., Barancik M. (2021). Oxidative stress and pathways of molecular hydrogen effects in medicine. Curr. Pharm. Des..

[90] Zhang Y., Zhao G., Cheng P., Yan X., Li Y., Cheng D., Wang R., Chen J., Shen W. (2019). Nitrite accumulation during storage of tomato fruit as prevented by hydrogen gas. Int. J. Food Prop..

[91] Seo T., Kurokawa R., Sato B. (2012). A convenient method for determining the concentration of hydrogen in water: Use of methylene blue with colloidal platinum. Med Gas Res..

[92] Murphy J.M., Sexton D.M., Jenkins G.J., Booth B.B., Brown C.C., Clark R.T., Collins M., Harris G.R., Kendon E.J., Betts R.A. (2009). UK Climate Projections Science Report: Climate Change Projections.

[93] Ray D.K., West P.C., Clark M., Gerber J.S., Prishchepov A.V., Chatterjee S. (2019). Climate change has likely already affected global food production. PLoS ONE.

[94] Dhankher O.P., Foyer C.H. (2018). Climate resilient crops for improving global food security and safety. Plant Cell Environ..

[95] Holden J., Haygarth P.M., Dunn N., Harris J., Harris R.C., Humble A., Jenkins A., MacDonald J., McGonigle D.F., Meacham T. (2017). Water quality and UK agriculture: Challenges and opportunities. Wiley Interdiscip. Rev. Water.

[96] Wu Q., Su N., Shabala L., Huang L., Yu M., Shabala S. (2020). Understanding the mechanistic basis of ameliorating effects of hydrogen rich water on salinity tolerance in barley. Environ. Exp. Bot..

[97] Cui W., Gao C., Fang P., Lin G., Shen W. (2013). Alleviation of cadmium toxicity in *Medicago sativa* by hydrogen-rich water. J. Hazard. Mater..

[98] Wu Q., Su N., Cai J., Shen Z., Cui J. (2015). Hydrogen-rich water enhances cadmium tolerance in Chinese cabbage by reducing cadmium uptake and increasing antioxidant capacities. J. Plant Physiol..

[99] Wu Q., Huang L., Su N., Shabala L., Wang H., Huang X., Wen R., Yu M., Cui J., Shabala S. (2020). Calcium-dependent hydrogen peroxide mediates hydrogen-rich water-reduced cadmium uptake in plant roots. Plant Physiol..

[100] Wu X., Su N., Yue X., Fang B., Zou J., Chen Y., Shen Z., Cui J. (2021). IRT1 and ZIP2 were involved in exogenous hydrogen-rich water-reduced cadmium accumulation in *Brassica chinensis* and *Arabidopsis thaliana*. J. Hazard. Mater..

[101] Chen M., Cui W., Zhu K., Xie Y., Zhang C., Shen W. (2014). Hydrogen-rich water alleviates aluminum-induced inhibition of root elongation in alfalfa via decreasing nitric oxide production. J. Hazard. Mater..

[102] Chen Q., Zhao X., Lei D., Hu S., Shen Z., Shen W., Xu X. (2017). Hydrogen-rich water pretreatment alters photosynthetic gas exchange, chlorophyll fluorescence, and antioxidant activities in heat-stressed cucumber leaves. Plant Growth Regul..

[103] Zhang X., Su N., Jia L., Tian J., Li H., Huang L., Shen Z., Cui J. (2018). Transcriptome analysis of radish sprouts hypocotyls reveals the regulatory role of hydrogen-rich water in anthocyanin biosynthesis under UV-A. BMC Plant Biol..

[104] Xie Y., Zhang W., Duan X., Dai C., Zhang Y., Cui W., Wang R., Shen W. (2015). Hydrogen-rich water-alleviated ultraviolet-B-triggered oxidative damage is partially associated with the manipulation of the metabolism of (iso) flavonoids and antioxidant defence in *Medicago sativa*. Funct. Plant Biol..

[105] Yeo A. (1998). Predicting the interaction between the effects of salinity and climate change on crop plants. Sci. Hortic..

[106] Sanders D. (2020). The salinity challenge. New Phytol..

[107] Kamran M., Parveen A., Ahmar S., Malik Z., Hussain S., Chattha M.S., Saleem M.H., Adil M., Heidari P., Chen J.T. (2020). An overview of hazardous impacts of soil salinity in crops, tolerance mechanisms, and amelioration through selenium supplementation. Int. J. Mol. Sci..

[108] Wani S.H., Kumar V., Khare T., Guddimalli R., Parveda M., Solymosi K., Suprasanna P., Kishor P.K. (2020). Engineering salinity tolerance in plants: Progress and prospects. Planta.

[109] Cole M.A., Elliott R.J., Shimamoto K. (2005). Industrial characteristics, environmental regulations and air pollution: An analysis of the UK manufacturing sector. J. Environ. Econ. Manag..

[110] Dixit S.S., Witcomb D. (1983). Heavy metal burden in water, substrate, and macroinvertebrate body tissue of a polluted River Irwell (England). Environ. Pollut. Ser. B Chem. Phys..

[111] Armitage P.D., Bowes M.J., Vincent H.M. (2007). Long-term changes in macroinvertebrate communities of a heavy metal polluted stream: The river Nent (Cumbria, UK) after 28 years. River Res. Appl..

[112] Nicholson F.A., Smith S.R., Alloway B.J., Carlton-Smith C., Chambers B.J. (2003). An inventory of heavy metals inputs to agricultural soils in England and Wales. Sci. Total Environ..

[113] Giusti L. (2011). Heavy metals in urban soils of Bristol (UK). Initial screening for contaminated land. J. Soils Sediments.

[114] Poschenrieder C., Gunsé B., Corrales I., Barceló J. (2008). A glance into aluminum toxicity and resistance in plants. Sci. Total Environ..

[115] Dixon E., Gardner M. (1998). Reactive aluminium in UK surface waters. Chem. Speciat. Bioavailab..

[116] Tipping E., Carter H.T. (2011). Aluminium speciation in streams and lakes of the UK Acid Waters Monitoring Network, modelled with WHAM. Sci. Total Environ..

[117] Xu Y., Ramanathan V., Victor D.G. (2018). Global warming will happen faster than we think. Nature.

[118] Diffey B. (2003). Climate change, ozone depletion and the impact on ultraviolet exposure of human skin. Phys. Med. Biol..

[119] Adesina O.S., Mohammed I. (2019). Potential impact of climate change on wheat production in Cambridgeshire, UK. J. Agric. Sci. Eng..

[120] IPCC Report. https://www.ipcc.ch/2021/08/09/ar6-wg1-20210809-pr/.

[121] McKenzie R.L., Aucamp P.J., Bais A.F., Björn L.O., Ilyas M., Madronich S. (2011). Ozone depletion and climate change: Impacts on UV radiation. Photochem. Photobiol. Sci..

[122] Bais A.F., McKenzie R.L., Bernhard G., Aucamp P.J., Ilyas M., Madronich S., Tourpali K. (2015). Ozone depletion and climate change: Impacts on UV radiation. Photochem. Photobiol. Sci..

[123] Jeswani H.K., Figueroa-Torres G., Azapagic A. (2021). The extent of food waste generation in the UK and its environmental impacts. Sustain. Prod. Consum..

[124] Hu H., Zhao S., Li P., Shen W. (2018). Hydrogen gas prolongs the shelf life of kiwifruit by decreasing ethylene biosynthesis. Postharvest. Biol. Technol..

[125] Jiang K., Kuang Y., Feng L., Liu Y., Wang S., Du H., Shen W. (2021). Molecular hydrogen maintains the storage quality of Chinese chive through improving antioxidant capacity. Plants.

[126] Chen H., Zhang J., Hao H., Feng Z., Chen M., Wang H., Ye M. (2017). Hydrogen-rich water increases postharvest quality by enhancing antioxidant capacity in *Hypsizygus marmoreus*. AMB Express.

[127] Huo J., Huang D., Zhang J., Fang H., Wang B., Wang C., Ma Z., Liao W. (2018). Comparative proteomic analysis during the involvement of nitric oxide in hydrogen gas-improved postharvest freshness in cut lilies. Int. J. Mol. Sci..

[128] Su J., Nie Y., Zhao G., Cheng D., Wang R., Chen J., Zhang S., Shen W. (2019). Endogenous hydrogen gas delays petal senescence and extends the vase life of lisianthus cut flowers. Postharvest Biol. Technol..

[129] Zhang J., Hao H., Chen M., Wang H., Feng Z., Chen H. (2017). Hydrogen-rich water alleviates the toxicities of different stresses to mycelial growth in *Hypsizygus marmoreus*. AMB Express.

[130] Hu H., Li P., Shen W. (2021). Preharvest application of hydrogen-rich water not only affects daylily bud yield but also contributes to the alleviation of bud browning. Sci. Hortic..

[131] Litalien A., Zeeb B. (2020). Curing the earth: A review of anthropogenic soil salinization and plant-based strategies for sustainable mitigation. Sci. Total Environ..

[132] Zulfiqar F., Russell G., Hancock J.T. (2021). Molecular hydrogen in agriculture. Planta.

[133] Singh S., Jain S., Venkateswaran P.S., Tiwari A.K., Nouni M.R., Pandey J.K., Goel S. (2015). Hydrogen: A sustainable fuel for future of the transport sector. Renew. Sustain. Energy Rev..

